# Unique structure of ozoralizumab, a trivalent anti-TNFα NANOBODY^®^ compound, offers the potential advantage of mitigating the risk of immune complex-induced inflammation

**DOI:** 10.3389/fimmu.2023.1149874

**Published:** 2023-04-14

**Authors:** Masanao Kyuuma, Ayaka Kaku, Chiemi Mishima-Tsumagari, Bunichiro Ogawa, Mayumi Endo, Yunoshin Tamura, Kei-ichiro Ishikura, Masashi Mima, Yutaka Nakanishi, Yasuyuki Fujii

**Affiliations:** Research Headquarters, Taisho Pharmaceutical Co., Ltd., Saitama, Japan

**Keywords:** tumor necrosis factor, VHH, rheumatoid arthritis, injection site reaction, immunogenicity, Fcγ receptor, anti-TNFα antibody, immune complex

## Abstract

Biologics have become an important component of treatment strategies for a variety of diseases, but the immunogenicity of large immune complexes (ICs) and aggregates of biologics may increase risk of adverse events is a concern for biologics and it remains unclear whether large ICs consisting of intrinsic antigen and therapeutic antibodies are actually involved in acute local inflammation such as injection site reaction (ISR). Ozoralizumab is a trivalent, bispecific NANOBODY^®^ compound that differs structurally from IgGs. Treatment with ozoralizumab has been shown to provide beneficial effects in the treatment of rheumatoid arthritis (RA) comparable to those obtained with other TNFα inhibitors. Very few ISRs (2%) have been reported after ozoralizumab administration, and the drug has been shown to have acceptable safety and tolerability. In this study, in order to elucidate the mechanism underlying the reduced incidence of ISRs associated with ozoralizumab administration, we investigated the stoichiometry of two TNFα inhibitors (ozoralizumab and adalimumab, an anti-TNFα IgG) ICs and the induction by these drugs of Fcγ receptor (FcγR)-mediated immune responses on neutrophils. Ozoralizumab-TNFα ICs are smaller than adalimumab-TNFα ICs and lack an Fc portion, thus mitigating FcγR-mediated immune responses on neutrophils. We also developed a model of anti-TNFα antibody-TNFα IC-induced subcutaneous inflammation and found that ozoralizumab-TNFα ICs do not induce any significant inflammation at injection sites. The results of our studies suggest that ozoralizumab is a promising candidate for the treatment of RA that entails a lower risk of the IC-mediated immune cell activation that leads to unwanted immune responses.

## 1 Introduction

Rheumatoid arthritis (RA) is one of a number of systemic autoimmune diseases that are accompanied by inflammation and both joint pain and swelling due to the inflammation. The roles of TNFα in the pathogenesis of RA have been reported (1, 2), and biologics that inhibit TNFα have made significant improvements in the treatment of RA (3–7). Five TNF inhibitors are now available for clinical use in RA, and the following four of them (the exception being certolizumab pegol) contain the Fc (Fragment crystallizable) portion: etanercept, a TNF receptor Fc fusion protein (3); infliximab, an anti-TNFα chimeric monoclonal antibody (4); adalimumab and golimumab, anti-TNFα human monoclonal antibodies (5, 6); and certolizumab pegol, a pegylated anti-TNFα humanized antibody Fab’ fragment (7).

The Fc portion of IgG antibodies mediates various immune response by interacting with the complement system or with Fcγ receptor (FcγR), which is widely expressed on several types of immune cells (dendritic cells, mast cells, neutrophils, monocytes, and macrophages). The effector functions of IgG antibodies, such as complement-dependent cytotoxicity (CDC) and antibody-dependent cellular cytotoxicity (ADCC), are thought to be important mechanisms underlying the actions of therapeutic antibodies aimed at cytotoxicity, such as rituximab and trastuzumab (8). However, these Fc-mediated responses also entail the risk of inducing various unwanted immune responses (9). Accumulating evidence has shown that the unwanted inflammatory response is closely associated with injection site reactions (ISRs) or systemic hypersensitivity reactions (10–12). These unwanted immune responses can be an important factor in deciding to discontinue biotherapy and in selecting biological therapeutics (13–15).

Cross-linking of FcγRs by multimerized IgG as a result of immune complex (IC) formation is known to be one of the triggers of these unwanted immune responses (9, 16, 17). The extent of FcγR-mediated activation has been reported to be correlated with IC size (18). The traditional IgG antibodies, such as adalimumab and infliximab, can generate extremely large ICs under certain conditions, because an IgG antibody interacts with only one TNFα molecule of TNFα trimers in the complex as the result of the low flexibility of IgG in the distance between two antigen binding domains (19, 20). Moreover, studies that used a reporter assay have found a correlation between the size of the ICs and FcγRII and FcγRIII receptor-downstream signaling (21), suggesting that ICs composed of anti-TNFα antibodies and TNFα would be capable of inducing an unwanted immune response. However, the results were obtained in studies that used FcγR-overexpressing cells, and no studies have ever been performed using primary cells directly isolated from the hematopoietic system. Moreover, it is unclear whether the activation of FcγR-expressing cells by ICs leads to acute inflammation at the local injection site.

The next-generation anti-TNFα therapeutic ozoralizumab is a 38kDa humanized trivalent NANOBODY^®^ compound that consists of two anti-human TNFα NANOBODY^®^ VHHs and an anti-human serum albumin (HSA) NANOBODY^®^ VHH, and it is structurally different from anti-TNFα IgGs such as adalimumab (22–24). Unlike previously reported IgG mAbs (19, 20), the very flexible geometry of three small NANOBODY^®^ VHHs fused together with a 9GS linker enables both anti-human TNFα NANOBODY^®^ VHHs to bind to two of the TNFα molecules of same TNFα trimers, and as a result ozoralizumab tends not to form large ICs with TNFα trimers. Moreover, since ozoralizumab lacks an Fc portion, ICs containing ozoralizumab might not be recognized by FcγRs expressed on immune cells. Consequently, ozoralizumab is expected to cause fewer immune responses caused by cross-linking of FcγRs with ICs. Treatment with ozoralizumab has been shown to have beneficial effects in the treatment of RA comparable to those of other TNF inhibitors and has been shown to have acceptable tolerability in Phase II/III clinical trials (25). The incidence of ISRs has been as low as 2%. The incidence of ISRs in the clinical studies of ozoralizumab has been similar to the incidence observed in clinical studies of certolizumab pegol (26), which also lacks an Fc portion, suggesting that the immune response *via* the Fc portion in antibody therapeutics is associated with the incidence of ISRs. In our previous paper, we demonstrated the immunogenicity assessment of biologics from biologics induce ADA production, indicating that the different structure of ozoralizumab compared to other TNF inhibitors is advantageous for the adaptive and systemic immune response induced by repeated administration (24). However, it remained unclear whether ozoralizumab is involved in localized acute inflammation.

In the present study, we therefore compared the effects of ICs formed with ozoralizumab or adalimumab (the latter as representative of antibodies that contain an Fc portion) on FcγR-mediated immune responses. We determined the molecular masses and hence stoichiometries of ICs by Size Exclusion Chromatography (SEC) and investigated whether ozoralizumab-TNFα ICs are capable of activating FcγR-expressing immune cells. We also developed a model of anti-TNFα antibodies and TNFα IC-induced subcutaneous inflammation to predict the potential clinical benefit of ozoralizumab in avoiding or reducing certain types of unwanted immune responses.

## 2 Materials and methods

### 2.1 Size exclusion chromatography

**Figure 1:**
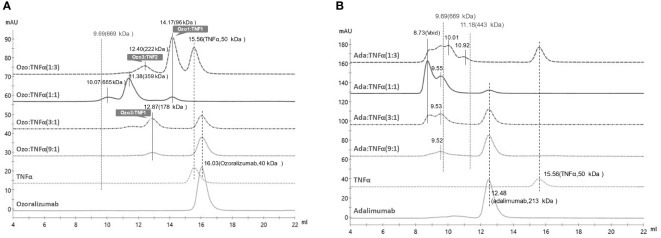
Size Exclusion Chromatography (SEC) of immunocomplexes of TNFα with ozoralizumab or adalimumab. **(A)** chromatograms of ozoralizumab-TNFα mixtures in various molar ratios. **(B)** chromatograms of adalimumab-TNFα mixtures in various molar ratios. All molecular weights were estimated by calibration with molecular weight markers.

Ozoralizumab or adalimumab (0.65 nmol) was mixed with 0.072, 0.217, 0.65, or 1.95 nmol of TNFα trimer in 250 μL of PBS. Each mixture was incubated overnight at 4°C and then loaded onto a Superdex 200 Increase 10/300 column equilibrated with PBS. Representative results from two independent experiments are shown in **Figure 1**. The molecular weight estimated by the elution volume at the column has been calibrated by using a Gel Filtration Calibration Kit (Cytiva, United States), Carbonic Anhydrase (29 kDa, Sigma, United States), Cytochrome C (12.4 kDa, Sigma, United States), and Aprotinin (6.5 kDa, Nakarai, Japan). The molecular masses of the complexes obtained were estimated by SEC Principles & Methods (Cytiva, United States). After mixing 0.65 nmol of ozoralizumab with 1.3 nmol HSA (Sigma-Aldrich, United States) and 0.072, 0.217, 0.65, or 1.95 nmol of TNFα trimer in 250 ul of PBS, each mixture was incubated overnight at 4°C and loaded onto a Superdex 200 Increase 10/300 column equilibrated with PBS. Molecular mass was estimated as described above.

### 2.2 *In silico* modeling and MD simulations

Molecular modeling and visualization of the TNFα-ozoralizumab complex were performed using the Molecular Operating Environment (MOE) 2019.01 software platform (27). The initial complex structure of the two anti-TNFα NANOBODY^®^ VHHs in ozoralizumab and hTNFα trimer was built based on the crystal structures of a TNFα and an anti-h TNFα NANOBODY^®^ VHH complex (PDB ID: 5M2J) (23). The structure of an anti-TNFα NANOBODY^®^ VHH ozoralizumab was modeled with a #VHH2 template by using the protein modeler tool in the “MOE/Homology Modeler” suite. The structure of an anti-HSA NANOBODY^®^ VHH was also modeled using the “MOE/Antibody Modeler” tools. The linkers (9 amide acids: GGGGSGGGS) between the anti-TNFα and anti-HSA NANOBODIES ^®^ were constructed by using the “MOE/Loop/Linker Modeler” tools. The structure (protonation, cap and build loop) for the crystal TNFα trimer structure was prepared with the “MOE/Structure Preparation” tools. In addition, the model structures were optimized by using the Amber10: EHT force field in generalized Born approximation, and all heavy atoms were fixed at the X-ray structure coordinates, except the two linkers. Molecular dynamics (MD) simulations of the ozoralizumab and TNFα trimer complex were then performed with GROMACS 2020.6 software (28) to confirm the validity of our predicted model. MD simulations were performed according to the procedure described below. The complex was placed in a truncated octahedron simulation box with a periodic boundary condition filled with TIP3P model water molecules (29). The water molecules were replaced with Na+ and Cl− ions to an NaCl concentration of 150 mM. The ff99SB force field parameter was assigned to the protein. To equilibrate the simulation system, a 20,000-step structural optimization and a 0.2 ns NVT MD simulation with position restraints for heavy atoms except water molecules were carried out. Then, the 0.2 ns MD without restraints under NPT conditions was continued. The temperature was maintained at 310 K and the pressure at 1 bar by using the stochastic dynamics algorithm (30) and the Berendsen algorithm (31), respectively. The long-range electrostatic interaction was processed by using the particle mesh Ewald method (32). An additional 0.2 ns MD simulation and a 100 ns production MD simulation were performed by applying the Nosé-Hoover and Parrinello-Rahman algorithm (33–35).

### 2.3 Isolation of primary mouse neutrophils

Mouse neutrophils were isolated from bone marrow by using a Neutrophil Isolation Kit (Cayman Chemical, United States) according to the manufacturer’s instructions. Neutrophils were isolated from blood at room temperature by using sterile, endotoxin-free reagents. Cells were kept at room temperature in CL medium (a modified RPMI 1640 medium without phenol red and sodium hydrogen carbonate [Merck, Germany] containing 20 mM HEPES [Thermo Fisher Scientific, United States]) as described previously (36) until used (usually within 60 min). Cells were pre-treated for 20 min with 50 ng/ml TNFα prior to IC stimulation and then incubated for 10 minutes with/without BUF041B, FcγRII/CD32, and RIII/CD16 neutralized antibodies.

### 2.4 Detection of reactive oxygen species

Ozoralizumab was generated at Wyeth Research in the manner previously described (37). Adalimumab was purchased from Eisai Co., Ltd (Japan). Recombinant human TNFα alpha was purchased from R&D Systems (United States). All samples were diluted with PBS (Thermo Fisher Scientific, United States) or saline (Otsuka, Japan). A 20.3 μM solution of ozoralizumab or adalimumab was mixed with 2.3 μM, 6.6 or 6.8 μM, 20.3 μM, or 60.8 μM solution of TNFα trimer and incubated overnight at 4°C. Mixtures of the respective antibodies and TNF were added to mouse neutrophils to a final antibody concentration of 33-34 nM. Reactive Oxygen Species (ROS) were measured by using L-012 sodium salt (8-Amino-5-chloro-2,3-dihydro-7-phenyl-pyrido[3,4-d] pyridazine sodium salt) (38). Neutrophils (5 x 105 cells/well) in CL medium containing 50 μM L-012 (FUJIFILM Wako Pure Chemical, Japan) were transferred to white microplates (Greiner Bio-One, Austria), and ROS-dependent chemiluminescence was measured with an ARVO microplate reader (PerkinElmer, United States). ROS released by the neutrophils were monitored for 1 min at 37°C.

### 2.5 Mice

Six-eight-week-old C57BL/6J male mice were purchased from Charles River Japan (Japan). The animals were housed under controlled temperature (23°C ± 3°C), humidity (55% ± 20%), and lighting (lights on from 0700 to 1900 hours) conditions. All animal experiments reported here were reviewed and approved by the Institutional Animal Care and Use Committee of Taisho Pharmaceutical Co., Ltd., and were in accordance with the Guidelines for Proper Conduct of Animal Experiments (Science Council of Japan, 2006).

### 2.6 Immune complex-induced subcutaneous inflammation

The dorsal skin of anesthetized mice was shaved, and 30 μL of PBS, ozoralizumab (6.6 μM, 0.25 mg/mL or 26.3 μM, 1 mg/mL), and adalimumab (6.8 μM, 1 mg/mL) mixed with 10 μg TNFα were injected intradermally at specific sites. Then, 0.5% Evans blue dye was immediately injected *via* the tail vein. The mice were sacrificed 4 hours after being injected, and the skin was removed at the fascia level above skeletal muscle, and reversed. Photographs were taken immediately and used to quantitate the degree of plasma exudation elicited by IC-induced subcutaneous inflammation. The blue dye intensity at each injection site was quantified with ImageJ software (National Institutes of Health, Bethesda, MD). The injection sites were then removed with a disposable sterile 6-mm punch biopsy (Kai, Japan), and after weighing them to evaluate edema, the tissues were immediately flash-frozen in liquid nitrogen and stored at −80°C.

### 2.7 Determination of MPO levels in skin

The flash-frozen skin tissues were blended and homogenized with RIPA Lysis and Extraction buffer (Thermo Fisher Scientific, United States) containing Halt Protease and Phosphatase Inhibitor (Thermo Fisher Scientific, United States). The homogenates were centrifuged at 10,000 g for 5 min, and the supernatant was collected. MPO levels in the tissue supernatant were measured by using a mouse ELISA kit (Abcam, United Kingdom) according to the manufacturer’s instructions.

### 2.8 Histopathology

The injection sites collected from the dorsal skin of the mice (n = 3/group) were fixed in 10% neutral buffered formalin fixation, trimmed, and embedded in paraffin. Hematoxylin and eosin stained slides were prepared, and they were examined histopathologically with a BX53; microscope (Olympus Corporation, Japan).

### 2.9 Determination of ADCC activity

Infliximab and adalimumab were locally purchased from Med World Pharmacy. 7AAD staining solution was purchased from BD Biosciences (United States). NS0-TNF-D13 cells were produced by transfecting NS0 cells with a plasmid encoding the membrane bound form of TNFα. NK cells were isolated from the blood of healthy donors by negative selection with the RosetteSep™ Human NK cell enrichment cocktail obtained from Stemcell Technologies (Canada). NS0-TNF-D13 cells labeled with CSFE were used as targets to assess ADCC activity. The assays were run in RPMI, 1% heat-inactivated FBS, 2 mM L-glutamine, 10 mM HEPES, 50 U/ml penicillin, and 50 μg/ml streptomycin. A 0.25 x 10^5^ number of NS0-TNF-D13 cells (targets cells) in 50 μl in a 96-well U-bottom plate was mixed with 50 μl of appropriately diluted anti-TNF agents or control antibody. After incubating the plates at 37°C for 20 min, effector cells were added at a target:effector ratio of at least 1:4, i.e., >0.12 x 10^6^ cells/well in 50 μl. The plates were then incubated for 4 hours at 37°C. 7AAD was added for the last 10 min, and the cells were acquired by flow cytometry. The flow cytometry data were analyzed with FlowJo software 7.2.2 (BD Biosciences, United States), and % ADCC activity was calculated by gating on the CSFE+ target cell population and calculating the percentage of cells that were 7AAD+. ADCC activity was determined at Wyeth Research, United States.

### 2.10 Determination of CDC activity

Infliximab, adalimumab, and etanercept were locally purchased from Med World Pharmacy. Sterile baby rabbit complement was purchased from Cedarlane Laboratories (Canada). Propidium Iodide staining solution was bought from BD Pharmingen (United States). The CHO- TNFα-NC2 cell line was produced by using CHO.DUKX cells that were transfected with a plasmid encoding a form of TNFα that is expressed both on the cell surface and in a secreted form. The cells were adapted to suspension media (CHO cell Media) containing R5CD1, 50 U/ml penicillin, 50 μg/ml streptomycin, 2 mM L-glutamine, and 20 nM Methotrexate and grown in a shaker flask at a 37°C in a shaker incubator under a 5% CO2 atmosphere. The plates were incubated in a 5% CO2 incubator for 3 hours 30 min at 37°C. To measure CDC activity, propidium iodide (6 μg/ml), a viability probe, was added to the cells, and after incubating them for 10 min at room temperature, 150 ul of PBS was added to all the wells. PI uptake was determined by using an HTS plate reader on a Becton-Dickinson LSR II flow cytometer, and the data were analyzed with FlowJo software 7.2.2 (BD Biosciences, United States). CDC activity was determined at Wyeth Research, United States.

### 2.11 Statistical analysis

Tukey’s post-test was used to compare the neutrophil activation chemiluminescence data. One-way ANOVA with Tukey’s post-test was used to compare the data obtained in the IC-induced subcutaneous inflammation model and to analyze differences in means among the groups.

## 3 Results

### 3.1 Differences in complex formation with TNFα between IgG antibody and trivalent NANOBODY^®^ compound

We previously demonstrated that ozoralizumab does not form large ICs in Ouchterlony double diffusion assays (24). However, since the stoichiometries of the ICs consisting of ozoralizumab and TNFα trimer are unclear, we evaluated the binding stoichiometry of ozoralizumab and adalimumab with TNFα trimer by SEC.

When ozoralizumab was pre-incubated with a threefold molar excess of TNFα trimers, SEC yielded three peaks. The major peak eluted at 14.17 ml (MW 96 kDa) was attributed to an ozoralizumab/TNFα trimer complex with a 1:1 stoichiometry, and the minor peak at 12.40 ml (MW around 222 kDa) was estimated to be an ozoralizumab/TNFα trimer complex with a 2:3 stoichiometry (three ozoralizumab molecules to two TNFα trimers) (**Figure 1A**). Unbound TNFα trimer was eluted at an elution volume of 15.56 ml (MW 50 kDa). When ozoralizumab and TNFα trimers were pre-incubated in equal molar ratios, two major peaks, at 11.38 ml (MW 359 kDa) and 10.07 ml (MW 665 kDa), attributed to heterogeneous ozoralizumab-TNFα ICs, and a small peak with an apparent MW of 96 kDa, attributed to an ozoralizumab/TNFα trimer complex with a 1:1 stoichiometry, were observed. When a threefold or ninefold molar excess of ozoralizumab was pre-incubated with TNFα, a peak at 12.87 ml (MW 178 kDa) attributed to an ozoralizumab/TNFα trimer complex with a 3:1 stoichiometry and a peak at 16.03 ml (MW 40 kDa) corresponding to unbound ozoralizumab were observed.

Similar experiments were performed with adalimumab. When adalimumab was pre-incubated with a threefold molar excess of TNFα trimers, broad peaks eluted ranging from 8.73 ml (void volume, MW ≥2000 kDa) to 10.92 ml (MW 443 kDa), attributed to heterogeneous sizes of adalimumab/TNFα trimer complexes, and a minor peak at 15.56 ml (MW 50 kDa) corresponding to TNFα trimer, were observed (**Figure 1B**). The ratio of the largest ICs was maximal when adalimumab and TNFα trimer were preincubated in equal molar ratios. The results demonstrated that ozoralizumab was unable to form large ICs eluted at void volume with TNFα at any molar ratio tested.

### 3.2 Structural *in silico* modeling and MD simulation of ozoralizumab and TNFα trimer complexes

As stated above, ozoralizumab is capable of forming a 1:1 complex with TNFα trimer under certain conditions. To confirm that one ozoralizumab is able to simultaneously bind to two TNFα in the TNFα trimer molecule, we predicted the 3-dimensional structure of the ozoralizumab-TNFα complex by in silico modeling and MD simulation. First, we modeled the structure of the TNFα - anti-TNFα NANOBODY^®^ VHH domain of the ozoralizumab complex by using the crystal structures of an IC between TNFα and - anti- TNFα NANOBODY^®^ VHH (#VHH2), whose amino acid sequence differs by 5 amino acids (PDB ID: 5M2J) (23). The 3-dimensional structure of the anti-HSA NANOBODY^®^ domain of ozoralizumab was then modeled in the same way with MOE software. Finally, the entire structure of ozoralizumab in which the two anti-TNFα NANOBODIES^®^ and anti-HSA NANOBODY^®^ VHH were connected with a 9-amino acid linker (GGGGSGGGS) was constructed by using the “MOE/Loop/Linker Modeler” tools.

**Figure 2:**
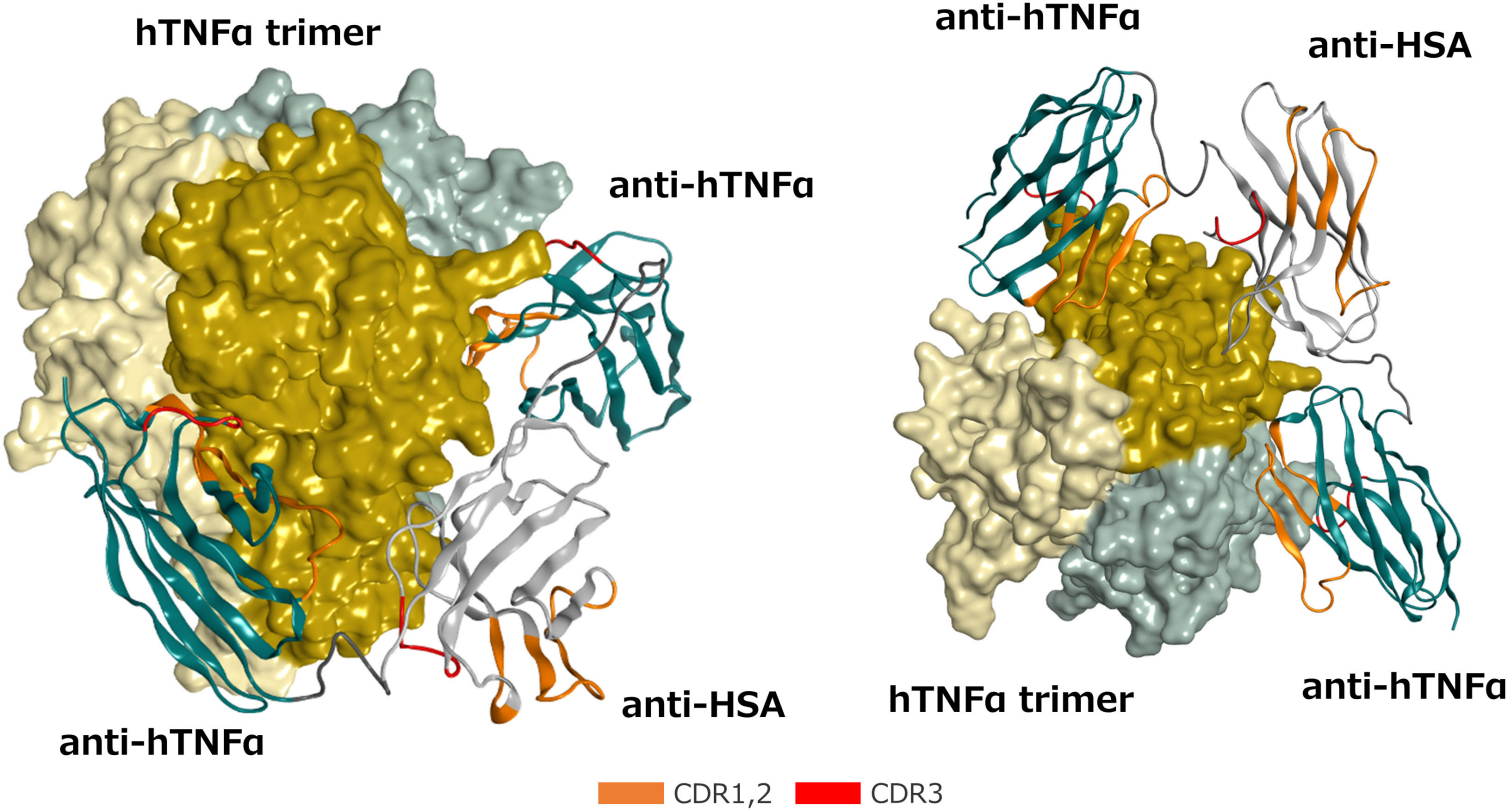
Front (Left) and bottom (Right) views of the predicted 3-dimensional structure of the ozoralizumab-TNFα trimer complex. The TNFα trimer molecule is shown with the molecular surface of each TNFα monomer unit colored dark yellow, light yellow, and light gray, respectively. The framework of the anti-TNFα NANOBODY^®^ and the framework of the anti-HSA NANOBODY^®^ are colored green and white, respectively. The CDRs are defined according to the Kabat numbering scheme and colored orange (CDR 1 and 2) and red (CDR3), respectively, and the two linkers are colored dark gray.

Since, based on the co-crystal structure of #VHH2 (23), these residues do not seem to be involved in the binding to the TNFα, it was considered appropriate to use #VHH2 as a template structure for in silico modeling of ozoralizumab TNFαNANOBODY^®^. We then performed MD simulations of the predicted TNFα-ozoralizumab complex structure to determine whether the 1:1 complex form was sufficiently stable. **Figure 2** shows the final snapshot of the 100 ns MD simulation of the TNFα-ozoralizumab complex. The average distance between the C-terminus of one anti-TNFα NANOBODY^®^ VHH and the N-terminus of the other anti-TNFα NANOBODY^®^ VHH was 58.1 angstrom (standard deviation: ± 5.9 angstrom), which seemed to be a reasonable length for the anti-HSA NANOBODY^®^ VHH located between the two anti-TNFα NANOBODIES^®^ by the amino acid linkers. The TNFα trimer — ozoralizumab complex we had modeled was maintained during the MD simulations described in Section 2 (Materials and Methods).

### 3.3 The Fc portion is indispensable to the generation of oxidative bursts in mouse neutrophils in response to antibody-TNFα IC-dependent stimuli

**Figure 3:**
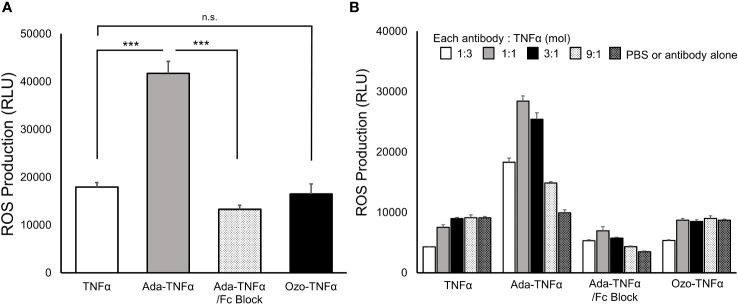
Production of ROS in mouse neutrophils activated by anti-TNFα antibody-TNFα ICs. Representative chemiluminescence values of extracellular MPO-dependent ROS as measured by the L-012 chemiluminescence assays are shown. ROS production by neutrophils (5 × 10^5^ cells/well in CL medium) after incubation for 1 min at 37°C with uncomplexed TNFα, adalimumab-TNFα ICs (Ada-TNFα), adalimumab-TNFα ICs with MOUSE SEROBLOCK FcR (Ada-TNFα/Fc Blocker), and ozoralizumab-TNFα ICs (Ozo-TNFα) was monitored. Equimolar amounts of anti-TNFα antibodies and TNFα were mixed for IC preparation in experiment **(A)**. In experiment **(B)**, ICs were prepared by adding anti-TNFα antibodies to TNFα in four different molar ratios. PBS, ozoralizumab, and adalimumab alone were used as controls in the experiments. Data are representative of three independent experiments, and values are expressed as the mean ± SEM. n.s., not significant; n=3 ^∗∗∗^p <0.001 vs. TNFα. (Tukey’s test).

Low-affinity FcγRs, IgG receptors that avidly bind to ICs, are key determinants of leukocyte activation (39). We evaluated oxidative bursts in mouse neutrophils in response to ozoralizumab-TNFα and adalimumab-TNFα, and the results confirmed the Fc portion - FcγRs contribution to anti-TNFα antibody-TNFα ICs-mediated stimulation. We isolated neutrophils from mouse bone marrow and incubated them with each of the TNFα inhibitor-TNFα ICs. Oxidative bursts were assessed by evaluating the production of mMPO-dependent ROS, which is considered a hallmark of neutrophil activation. Adalimumab-TNFα ICs significantly induced ROS production by mouse neutrophils *in vitro*, but no ROS production was induced by ozoralizumab-TNFα ICs under the same conditions. In parallel studies, mouse neutrophil activation by stimulation with adalimumab-TNFα ICs was abrogated by the inclusion of blocking antibodies against both FcγRII/CD32 and RIII/CD16 (**Figure 3A**).

Large ICs have been reported to show stronger affinity for low-affinity FcγRs than smaller ICs do (18). Since the stoichiometry of ICs varies with the antigen-antibody molar ratio, each TNF inhibitor was mixed with TNFα in different molar ratios ([TNFα trimer]:[TNF inhibitor] = 1:3, 1:1, 3:1, and 9:1) and assessed for neutrophil activation. Marked ROS production was observed with the adalimumab-TNFα ICs at all molar ratios, and maximum activation occurred at the 1:1 molar ratio. ROS production in response to all adalimumab-TNFα IC molar ratios tested was completely suppressed by the inclusion of FcγRII/CD32 and RIII/CD16 blocking antibodies (**Figure 3B**). Expectedly, no ROS production was observed with the ozoralizumab-TNFα ICs under any of the conditions tested. The results suggest that the neutrophil activation induced by the TNFα inhibitor-TNFα ICs was caused by the interaction between the Fc portion of TNFα inhibitor and low-affinity FcγRs. While ROS production that results from cross-linking of Fc receptors differs depending on the stoichiometry of the ICs, the Fc-FcgR interaction is, however, critical for this response.

### 3.4 Administration of ozoralizumab-TNFα ICs did not induce a significant inflammatory response in a model of IC-induced subcutaneous inflammation

ISRs are the major adverse events reported after the administration of TNF inhibitors (10, 11). Although ISRs are a type of hypersensitivity reaction caused by an excessive immune response (11), the contribution of IC formed between TNFα trimers and TNF antibodies to the occurrence of such a reaction is unclear. We therefore investigated whether intradermally administered ozoralizumab-TNFα ICs and adalimumab-TNFα ICs would induce topical inflammation in animals. Vascular permeability and plasma neutrophil extravasation, which are hallmarks of the IC-induced immunopathologic cascade, can be visualized by the extravasation of intravenously injected Evan blue dye and quantified by tissue weight.

**Figure 4:**
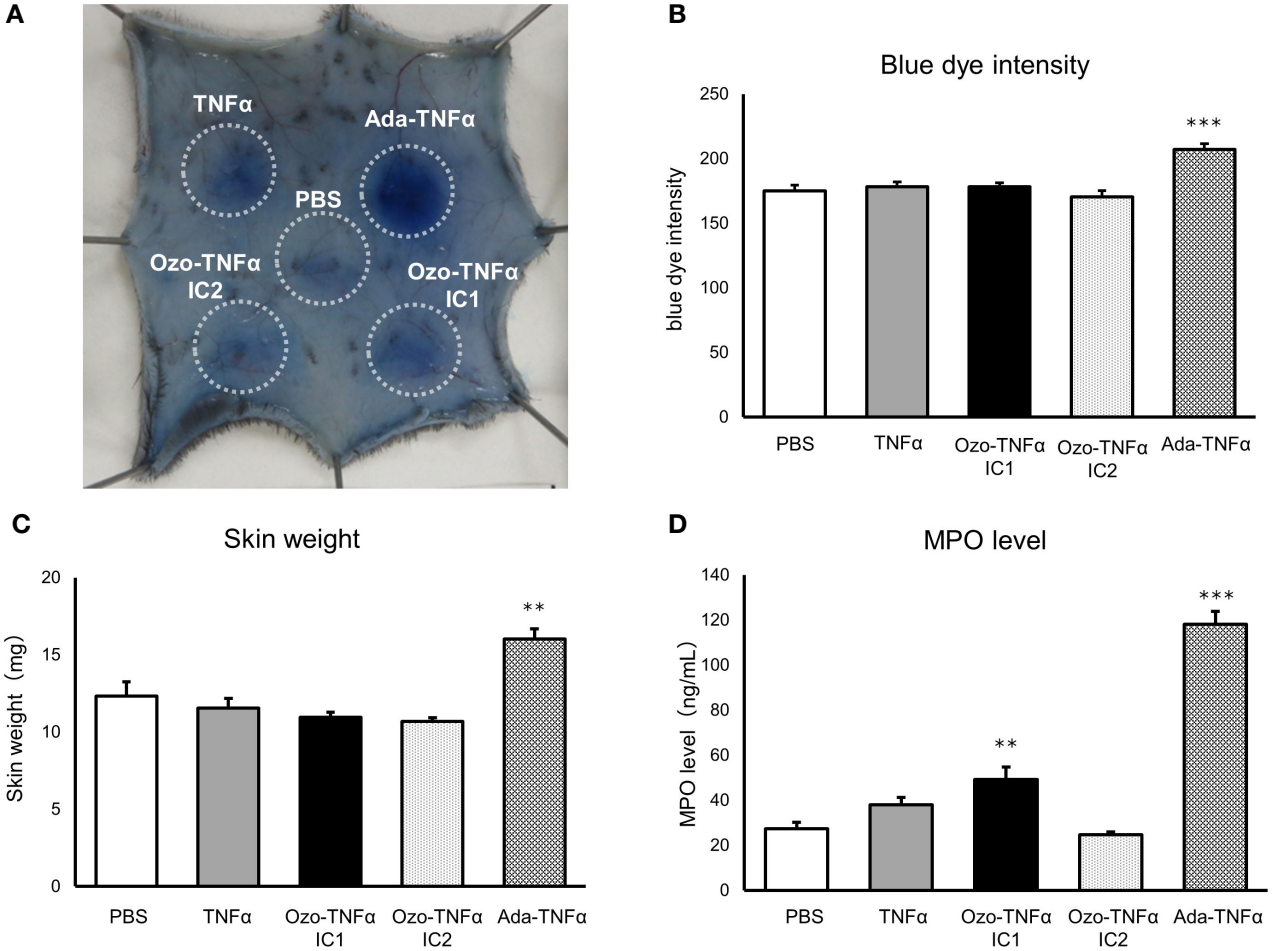
Inflammatory responses in skin tissue injected with pre-incubated ICs. C57BL/6J mice were intradermally injected with PBS, TNFα, ozoralizumab-TNFα IC1 (Ozo-TNFα IC1), ozoralizumab-TNFα IC2 (Ozo-TNFα IC2), or adalimumab-TNFα ICs (Ada-TNFα ICs). Degree of plasma exudation was evaluated by monitoring after intravenous injection of 0.5% Evans blue. **(A)** Representative macroscopic appearance of the subcutaneous inflammation. The circles surround the exuded Evans blue dye at each injection site. **(B)** Blue dye intensity quantified with ImageJ software. **(C)** Weight of 6-mm skin punch biopsy specimens. **(D)** MPO levels in tissue supernatant extracted from the 6-mm skin punch biopsy specimens. Data are expressed as mean ± SE. n = 7-8 ^∗∗^p <0.01, ^∗∗∗^p <0.001 vs. PBS (Tukey’s test).

To maximize the size of the ICs, ozoralizumab (6.6 μM, 0.25 mg/mL: ozoralizumab-TNFα IC1) and adalimumab (6.8 μM, 1 mg/mL) were mixed with equimolar TNFα (6.5 μM), and ozoralizumab (26.3 μM, 1 mg/mL: ozoralizumab-TNFα IC2) was mixed with 6.5 μM of TNFα to evaluate the size dependency of the ICs for topical inflammation. After pre-incubation, these ICs or PBS was intradermally injected into mice. The blue dye intensity was significantly higher at sites injected with adalimumab-TNFα ICs (P < 0.001) than at the sites injected with PBS (**Figures 4A, B**), whereas the blue dye intensity at the sites injected with ozoralizumab-TNFα IC1 and 2 was almost the same as at the sites injected with PBS (**Figures 4A, B**). Similar results were obtained when the wet weight of skin biopsy specimens from IC injection sites were measured as an indicator of edema (**Figure 4C**). The skin injected with adalimumab-TNFα ICs weighed significantly more (P < 0.001) than the skin injected with PBS (**Figure 4C**). By contrast, no weight changes were observed in the skin injected with ozoralizumab-TNFα IC 1 or 2, or with TNFα alone. No changes in dye intensity or skin weight were observed at the sites injected with antibody alone (**Supplementary Figure 1**). These results indicated adalimumab-TNFα ICs, which can form large ICs containing the Fc portion, induced edema and vascular hyperpermeability at the injection site, but the ozoralizumab-TNFα ICs did not. It is noteworthy that the adalimumab-TNFα ICs had sufficient ability to induce a local response, whereas similarly administered ozoralizumab-TNFα ICs did not induce any significant local immune response.

### 3.5 Ozoralizumab-TNFα ICs dose MPO induction, but do not induce any marked neutrophil infiltration at injection sites

MPO is a peroxidase enzyme that is expressed most abundantly in neutrophil granules, and it is a critical component of the phagocytic microorganism-killing activities of the innate immune system (40). Measuring MPO in the skin is a well-established method of estimating its neutrophil content in cutaneous inflammation (41, 42). In this study we evaluated neutrophil accumulation by measuring the MPO content of skin punches by an ELISA. The results showed that the local MPO concentration in the skin 4 hours after injection with adalimumab-TNFα ICs was markedly higher (118.22 ± 5.66 ng/mL) than in the skin injected with PBS (27.34 ± 2.99 ng/mL) (**Figure 4D**). By contrast, ozoralizumab TNFα IC2-injected sites or TNFα-injected sites were not significantly different from PBS-injected sites (24.65 ± 1.32 ng/mL and 37.94 ± 3.34 ng/mL, respectively). Local MPO concentrations at the ozoralizumab TNFα IC1 injection site (49.15 ± 5.62 ng/mL) were significantly different from PBS injection site and lower than local MPO concentrations at the adalimumab injection site. Since ozoralizumab TNFα IC2 is thought to form smaller ICs than ozoralizumab TNFα IC1, the results indicate that MPO level is dependent on the size of the ICs.

**Figure 5:**
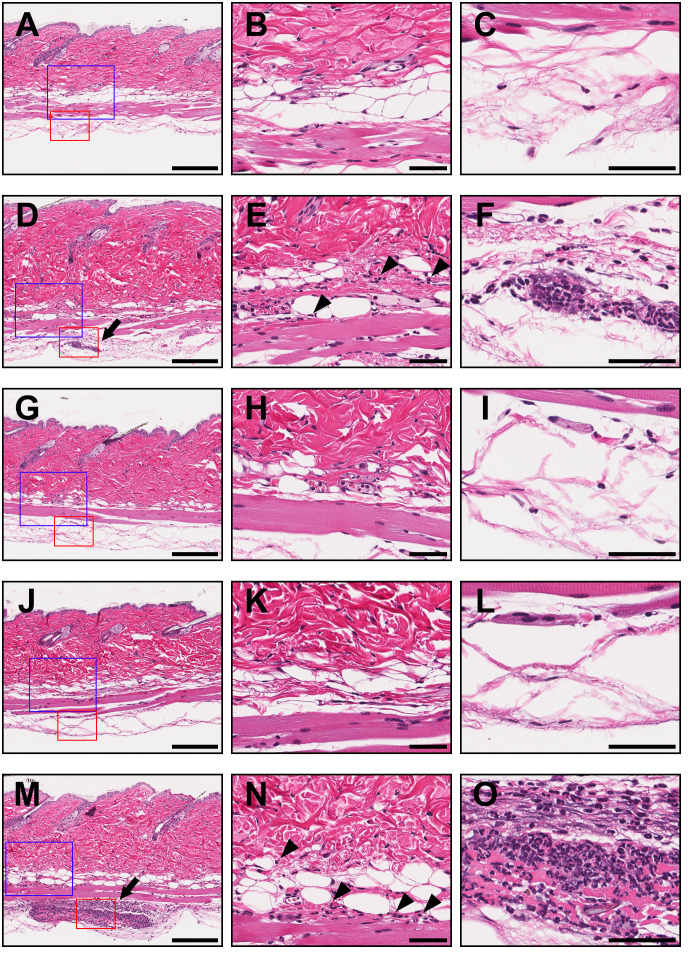
Histopathological examination of injection sites in a mouse model of IC-induced subcutaneous inflammation. The skin biopsy specimens collected from injected areas of PBS **(A–C)**, TNFα **(D–F)**, ozoralizumab-TNFα IC1 **(G–I)**, ozoralizumab-TNFα IC2 **(J–L)**, and adalimumab-TNFα ICs **(M–O)** were examined histopathologically after hematoxylin and eosin staining. The center column and right column are high magnification views of the blue boxes and red boxes, respectively, in the left column. The arrows point to focal neutrophil infiltration in the deeper layer of the subcutaneous tissue in the TNFα **(D)** and adalimumab-TNFα IC **(M)** injected areas. Neutrophils had also sporadically infiltrated the superficial layer of subcutaneous tissue (arrowheads) in the TNFα **(E)** and adalimumab-TNFα IC **(N)** injected areas. By contrast, the areas injected with ozoralizumab-TNFα IC1 **(G–I)**, ozoralizumab-TNFα IC2 **(J–L)**, and PBS **(A–C)** did not show any evidence of inflammatory cell infiltration. Scale bars: 200 µm (left), 50 µm (center and right).

To visualize the intensity and characteristics of the inflammation in response to the IC challenges, the skin sections taken from each group of mice were subjected to histopathological examination (**Figure 5**, **Table 1**). Skin sections from the adalimumab-TNFα IC-injected mice revealed an inflammatory cell infiltration predominantly composed of neutrophils, recognized by their multilobulated nuclei (**Figures 5M–O**), and representative pictures demonstrated that higher numbers of neutrophils had been recruited than in the mice injected with PBS (**Figures 5A–C**) or TNFα (**Figures 5D–F**). The arrows in the photomicrographs indicate focal neutrophil infiltration in the subcutaneous tissue at an adalimumab-TNFα IC-injected site (**Figure 5M**). By contrast, the skin sections from the mice injected with ozoralizumab-TNFα IC1 (**Figures 5G–I**) or ozoralizumab-TNFα IC2 (**Figures 5J–L**) did not show evidence of inflammatory cell infiltration, nor did the skin sections from the PBS-injected mice (**Figures 5A–C**). These histopathological findings confirmed the results obtained by the dye extravasation method (**Figure 4**), suggesting that, adalimumab-TNFα ICs are capable of triggering an IC-induced immune response accompanied by neutrophil infiltration, but that ozoralizumab-TNFα ICs are not.

**Table 1:** Histopathologic findings in a mouse model of immune complex (IC)-induced subcutaneous inflammation.

Groups	PBS			TNFα			Ozoralizumab-TNFα IC1			Ozoralizumab-TNFα IC2			Adalimumab-TNFα ICs		
Number of animals	3			3			3			3			3		
Grade	–	±	+	–	±	+	–	±	+	–	±	+	–	±	+
Skin (injection site)															
Subcutaneous neutrophil infiltration	3	0	0	2	1	0	3	0	0	3	0	0	0	1	2

-, None/Negligible, ±, Minimal/Very slight, +, Mild/Slight.

ICs, immune complexes; PBS, phosphate-buffered saline; TNF, tumor necrosis factor.

## 4 Discussion

The first step in the immune response is recognition of a foreign substance by tissue-resident innate immune cells, such as by mast cells and macrophages sensing ICs by their FcγRs, complement receptors and pattern recognition receptors (PRR) (43, 44), which results in the production of secondary mediators such as TNFα and chemokines, and subsequently promotes neutrophil infiltration (45). Large ICs consisting of intrinsic antigen and therapeutic antibodies are more likely to be recognized by FcγRs on the surface of innate immune cells, and they can induce immune responses *in vitro*, such as the secretion of pro-inflammatory cytokines and chemokines (46). However, it is not clear whether these ICs consisting of anti-TNFα antibodies and TNFα can induce acute inflammation at local site.

The properties of the target molecules are also important to the formation of large ICs. Theoretically, large ICs can be formed only by a combination of multimeric antigen and multivalent antibodies. TNFα forms a trimer and TNFα trimers tend to be to form large ICs with bivalent antibodies. However, as demonstrated in **Figure 1**, ozoralizumab did not form large ICs eluted at void volume in the SEC under any of the conditions tested; in contrast, adalimumab did form them under certain conditions. The difference may be attributable to the TNFα trimer binding styles of the IgG antibody and ozoralizumab. It may be impossible for conventional IgGs to bind to two TNFα molecules in the same TNFα trimer with its two antigen-binding sites because of the large size of Fab and limited flexibility of the hinges. Actually, no elution peaks corresponding to adalimumab-TNFα trimer (1:1) complexes were observed under any of the conditions tested (**Figure 1B**). Thus, anti-TNFα IgGs are naturally prone to form larger ICs by cross-linking two TNFα trimers. On the other hand, as shown in **Figure 2**, ozoralizumab was well designed to be able to capture two TNFα molecules in the same TNFα trimer with its two anti-TNFα NANOBODY^®^ VHHs, which have flexibility linkers, resulting in improved binding affinity and neutralizing activity (23, 24). Indeed, ozoralizumab-TNFα trimer (1:1) complexes were observed under certain conditions (**Figure 1A**). Ozoralizumab contains an anti-HSA NANOBODY^®^ VHH that can bind to HSA, but it was unclear whether ozoralizumab/TNFα/HSA heterotrimers are formed. After addition of a 2-fold molar excess of HSA, the ozoralizumab/TNF IC elution peak and single ozoralizumab elution peak were no longer seen, and an ozoralizumab/TNFα/HSA heterotrimer peak corresponding to HSA binding was observed under all conditions (**Supplementary Figure 2**). Interestingly, the heterogeneous ozoralizumab-TNFα IC (Ozo ^n^: TNFα trimers ^n^) peak at 11.38 ml (MW 359 kDa) observed in the absence of HSA (**Figure 1A**, Ozo : TNFα (1:1)) was no longer seen, and a dominant peak with an apparent MW of 250 kDa attributed to ozoralizumab/TNFα/HSA complexes with a 1:1:1 stoichiometry was observed (**Supplementary Figure 2**, Ozo : TNFα [1:1]). The results demonstrated that ozoralizumab binding to HSA did not inhibit binding to TNFα, but may instead reduce the formation of high-order ICs. Thus, because of its unique structure, ozoralizumab appears to be superior to other anti-TNFα IgG antibodies which tend to form larger ICs.

A minimal threshold of interaction between ICs and low affinity FcγRs is required for FcγR-expressing cell activation. In our *in vitro* experiment, adalimumab-TNFα ICs significantly induced ROS production by mouse neutrophils, whereas no ROS production was induced by ozoralizumab-TNFα ICs under the same conditions. The difference between the induction of ROS production by the two ICs suggested a difference between the interactions of the two ICs and FcγRs on neutrophils. The ROS induction by adalimumab-TNFα ICs was completely abrogated by the addition of anti-FcγRII and anti-FcγRIII neutralizing antibodies, and the ozoralizumab-TNFα ICs, which lack an Fc portion, did not induce ROS production (**Figure 3**). These findings suggest the important role of the Fc portion in adalimumab in neutrophil activation *via* cross-linking of FcγRs. As shown in **Figure 1B** and previously reported (18, 47), adalimumab is able to form large ICs with TNFα trimers (≥2000kDa) under certain conditions. The neutrophil activation by adalimumab-TNFα ICs (**Figure 3B**) seemed to be correlated with the amounts of large ICs (**Figure 1B**). Adalimumab-TNFα ICs prepared from equimolar amounts of the antibodies and TNFα trimers have been reported to induce maximal FcγRII- and FcγRIII-mediated cell signaling, as shown in reporter cell lines (21), a finding that is in consistent with the findings in our studies using primary cells. The small ICs formed by ozoralizumab do not establishe the threshold between ICs and low affinity FcγRs, thus indicating that ozoralizumab-ICs are not part of the FcγR-mediated activation. In addition, did not induce ADCC, one of the effector responses that are regulated by FcγRs (**Supplementary Figure 3**). Taken together ozoralizumab could be freed from the unwanted immune response of the FcγR function.

We developed a model of anti-TNFα antibodies and TNFα IC-induced subcutaneous inflammation and demonstrated that adalimumab-TNFα ICs can induce acute subcutaneous inflammation at injection sites in animals, but that ozoralizumab-TNFα ICs cannot (**Figures 4A–C**). FcγRIII-deficient mice, which lack IgG-mediated mast cell degranulation, are resistant to IgG-dependent passive cutaneous anaphylaxis, and exhibit an impaired Arthus reaction, which suggests the importance of FcγRs in the IC-mediated immune response (48). The FcγR-mediated signaling deficiency led to a reduction in extravasation and neutrophil accumulation of at the inflammation site, consistent with our observations. Ozoralizumab-TNFα ICs did not induce increased vascular permeability and plasma extravasation, which were visualized with Evans blue dye (**Figures 4A–C**). The complement system is known to be involved in vascular permeability and plasma extravasation, and complement depletion with cobra venom factor has been shown to abrogate extravasation in FcγIII α chain–deficient mice (48, 49). Ozoralizumab did not cause CDC in an assay using transmembrane TNFα-expressing CHO- TNFα-NC2 cell line as target cells and baby rabbit complement, although adalimumab, infliximab, and etanercept did (**Supplementary Figure 3**). Unexpectedly, ozoralizumab-TNFα IC1 induced slight neutrophil recruitment based on the MPO levels (**Figure 4D**), suggesting that they were sensed by tissue-resident innate immune cells *via* another mechanism besides FcγRs. Ozoralizumab-TNFα IC2, which contains more ozoralizumab, did not induce any marked MPO induction, indicating that the sensing was not due to recognition of the foreign sequence in ozoralizumab. The sensing of biologic aggregation by PRRs, such as TLRs, is known to lead to significant increases in inflammatory cytokine secretion (50, 51). Ozoralizumab-TNFα ICs may be sensed by innate immune systems *via* PRRs. It has been suggested that the immune response activation by aggregated biologics depends on the number of particles and their surface area (52). Since IC size decreases under antibody excess conditions (**Figure 1**), the difference between the ozoralizumab-TNFα IC1 and ozoralizumab-TNFα IC2 immune responses suggests that the size of ICs may contribute to the Fc portion-independent immune response. Despite no neutrophil recruitment was seen in the histopathological examination (**Figure 5**), the increased MPO levels seen in **Figure 4D**. The increase in MPO levels in local injection site may have been induced by immune cells such as macrophages; it is possible that the immune response seen with ozoralizumab-TNFα IC1 administration was not sufficient to recruit neutrophils and subsequently not have led to the induce inflammatory symptoms such as extravasation and edema. These results indicated that ICs composed of anti-TNFα antibodies and TNFα ould induce acute inflammation at the local injection site, and therefore ozoralizumab, which does not interact with complement and does not cross-link FcγR is easy to operate biologics that is free from the acute inflammation caused by the FcγR and complement-related effector functions.

Our findings suggest that differences between the characteristics of ICs formed by different antibody formats can partially explain the different incidences of ISRs observed during treatment with anti-TNFα antibodies (**Figures 4**, **5**) (10, 11, 25, 26). The administered biologics diffuse from the site of administration (subcutaneous tissue) and are absorbed through capillaries and transferred into the bloodstream. During these diffusion processes, microscopically, they are thought to undergo antigen-antibody ratios that tend to form large ICs, such as those shown in the SEC. Large ICs are recognized by tissue-resident innate immune cells and complement, leading to neutrophil recruitment. In addition, the deposition of ICs under the subcutaneous tissue and capillary endothelium can exacerbate further inflammation. These reactions are a kind of type III hypersensitivity reactions (ICs-mediated reaction) and can cause symptoms of ISRs. Indeed, ISRs were reported in a small proportion of patients treated with ozoralizumab (2%) and with certolizumab pegol (4.3%), which like ozoralizumab lacks the Fc portion and forms small ICs. Taken together, these findings suggest that biologics without Fc portion and with small ICs formation capacity has shown the potential to lead to a mitigation in local acute inflammation.

In summary, unlike adalimumab, ozoralizumab mitigates FcγR‐mediated immune responses on neutrophils and does not induce any significant inflammation at injection sites. The differences were most likely attributable to the size of ICs and involvement of the Fc portion. Based on our finding, biologics without Fc portion and with low immune complex formation capacity has shown the potential to lead to a mitigation in local hypersensitivity reactions. Therefore, ozoralizumab mitigates the risk of acute inflammation, which may be reflected in the low incidence of ISRs. The unique structure of ozoralizumab provide a beneficial treatment for RA patients.

## Data availability statement

The original contributions presented in the study are included in the article/**Supplementary Material**. Further inquiries can be directed to the corresponding author.

## Ethics statement

All experimental procedures involving animal handling were approved by the Institutional Animal Care and Use Committee of Taisho Pharmaceutical Co., LTD., and were in accordance with the Guidelines for Proper Conduct of Animal Experiments (Science Council of Japan, 2006).

## Author contributions

MK, and YF contributed to conception and design of the study. MK, AK and KI performed the *in vivo* experiments. MK, CT and MM performed the *in vitro* experiments. BO and YN performed the histopathology. ME and YT organized the *in silico* modeling. MK wrote the first draft of the manuscript. AK, BO, ME, MM, and YN wrote sections of the manuscript. All authors contributed to the article and approved the submitted version.

## Supplementary material

Supplementary Figure 1 Inflammatory responses in the skin of mice to intradermally injected with anti TNFα antibody alone and pre-incubated ICs. Mice were intradermally injected with PBS, ozoralizumab-TNFαICs (Ozo-TNFα IC1, the same conc/ratio as in **Figure 4**), adalimumab-TNFαICs (Ada-TNFα ICs, the same conc/ratio as in **Figure 4**), ozoralizumab (Ozo) and adalimumab (Ada) and then intravenously injected with 0.5% Evans blue. **(A)** Representative macroscopic appearance of the subcutaneous inflammation. The circles surround exuded Evans blue dye at each injection site. **(B)** Blue dye intensity was quantified with ImageJ software. **(C)** Skin weight of 6-mm skin punch biopsy specimens. n = 6 *** p <0.001 vs. PBS (Tukey’s test).

Supplementary Figure 2 Size Exclusion Chromatography (SEC) of immunocomplexes of ozoralizumab-TNFα-HSA. All molecular weights were estimated by calibration with molecular weight markers.

Supplementary Figure 3
**(A)** ADCC activity of infliximab, adalimumab, and a control IgG1 antibody (a human IgG1 antibody raised against the HIV envelop) were compared with ozoralizumab by using CFSE-labeled NS0-TNFD13 cells as targets and human NK cells as effectors. % ADCC activity values were calculated as the percentage of target cells that were 7AAD^+^. **(B)** CDC activity of etanercept, adalimumab, infliximab, and a control IgG1 antibody (a human IgG1 antibody raised against the HIV envelope) was compared with the CDC activity of ozoralizumab on the CHO-TNF NC2 line *in vitro* in the presence of baby rabbit complement. Cytotoxicity was assessed by measuring PI uptake of the dead cells. Values plotted are percentages of PI+ cells with the test and control subtracted from % PI- cells in the presence of complement alone.

## References

[1] Scott DL Wolfe F Huizinga TW. Rheumatoid arthritis. Lancet (2010) 376(9746):1094–108. doi: 10.1016/S0140-6736(10)60826-4 20870100

[2] McInnes IB Schett G. Pathogenetic insights from the treatment of rheumatoid arthritis. Lancet (2017) 389(10086):2328–37. doi: 10.1016/S0140-6736(17)31472-1 28612747

[3] Moreland LW Baumgartner SW Schiff MH Tindall EA Fleischmann RM Weaver AL. Treatment of rheumatoid arthritis with a recombinant human tumor necrosis factor receptor (P75)-fc fusion protein. N Engl J Med (1997) 337(3):141–7. doi: 10.1056/NEJM199707173370301 9219699

[4] Elliott MJ Maini RN Feldmann M Kalden JR Antoni C Smolen JS. Randomised double-blind comparison of chimeric monoclonal antibody to tumour necrosis factor α (cA2) versus placebo in rheumatoid arthritis. Lancet (1994) 344(8930):1105–10. doi: 10.1016/s0140-6736(94)90628-9 7934491

[5] Weinblatt ME Keystone EC Furst DE Moreland LW Weisman MH Birbara CA. Adalimumab, a fully human anti-tumor necrosis factor alpha monoclonal antibody, for the treatment of rheumatoid arthritis in patients taking concomitant methotrexate: the ARMADA trial. Arthritis Rheumatol (2003) 48(1):35–45. doi: 10.1002/art.10697 12528101

[6] Zhou H Jang H Fleischmann RM Bouman-Thio E Xu Z Marini JC. Pharmacokinetics and safety of golimumab, a fully human anti-TNF-Alpha monoclonal antibody, in subjects with rheumatoid arthritis. J Clin Pharmacol (2007) 47(3):383–96. doi: 10.1177/0091270006298188 17322150

[7] Choy EHS Hazleman B Smith M Moss K Lisi L Scott DGI. Efficacy of a novel PEGylated humanized anti-TNF fragment (CDP870) in patients with rheumatoid arthritis: A phase II double-blinded, randomized, dose-escalating trial. Rheumatol (Oxf) (2002) 41(10):1133–7. doi: 10.1093/rheumatology/41.10.1133 12364632

[8] Clynes RA Towers TL Presta LG Ravetch JV. Inhibitory fc receptors modulate in vivo cytoxicity against tumor targets. Nat Med (2000) 6(4):443–6. doi: 10.1038/74704 10742152

[9] Nimmerjahn F Ravetch JV. Fcγ receptors as regulators of immune responses. Nat Rev Immunol (2008) 8(1):34–47. doi: 10.1038/nri2206 18064051

[10] Matsui T Umetsu R Kato Y Hane Y Sasaoka S Motooka Y. Age-related trends in injection site reaction incidence induced by the tumor necrosis factor-α (TNF-α) inhibitors etanercept and adalimumab: the food and drug administration adverse event reporting system, 2004-2015. Int J Med Sci (2017) 14(2):102–9. doi: 10.7150/ijms.17025 PMC5332837 28260984

[11] Murdaca G Spanò F Puppo F. Selective TNF-α inhibitor-induced injection site reactions. Expert Opin Drug Safety (2013) 12(2):187–93. doi: 10.1517/14740338.2013.755957 23330811

[12] Leach MW Rottman JB Hock MB Finco D Rojko JL Beyer JC. Immunogenicity/Hypersensitivity of biologics. Toxicol Pathol (2014) 42(1):293–300. doi: 10.1177/0192623313510987 24240973

[13] Böhm R Proksch E Schwarz T Cascorbi I. Drug hypersensitivity: Diagnosis, genetics, and prevention. Deutsches Ärzteblatt Int (2018) 115:501–12. doi: 10.3238/arztebl.2018.0501 PMC6121083 30135011

[14] Sauna ZE Lagassé D Pedras-Vasconcelos J Golding B Rosenberg AS. Evaluating and mitigating the immunogenicity of therapeutic proteins. Trends Biotechnol (2018) 36(10):1068–84. doi: 10.1016/j.tibtech.2018.05.008 29908714

[15] Barton J. Patient preferences and satisfaction in the treatment of rheumatoid arthritis with biologic therapy. Patient Preference Adherence (2009) 3:335. doi: 10.2147/ppa.s5835 20016797 PMC2792871

[16] Bournazos S Gupta A Ravetch JV. The role of IgG fc receptors in antibody-dependent enhancement. Nat Rev Immunol (2020) 20(10):633–43. doi: 10.1038/s41577-020-00410-0 PMC7418887 32782358

[17] Krishna M Nadler SG. Immunogenicity to biotherapeutics the role of anti-drug immune complexes. Front Immunol (2016) 7:21. doi: 10.3389/fimmu.2016.00021 26870037 PMC4735944

[18] Lux A Yu X Scanlan CN Nimmerjahn F. Impact of immune complex size and glycosylation on IgG binding to human FcγRs. J Immunol (2013) 190(8):4315–23. doi: 10.4049/jimmunol.1200501 23509345

[19] Kohno T Tam LT Stevens SR Louie JS. Binding characteristics of tumor necrosis factor receptor-fc fusion proteins vs anti-tumor necrosis factor MAbs. J Invest Dermatol Symposium Proc (2007) 12(1):5–8. doi: 10.1038/sj.jidsymp.5650034 17502862

[20] Scallon B Cai A Solowski N Rosenberg A Song X Shealy D. Binding and functional comparisons of two types of tumor necrosis factor antagonists. J Pharmacol Exp Ther (2002) 301(2):418–26. doi: 10.1124/jpet.301.2.418 11961039

[21] Krayukhina E Noda M Ishii K Maruno T Wakabayashi H Tada M. Analytical ultracentrifugation with fluorescence detection system reveals differences in complex formation between recombinant human TNF and different biological TNF antagonists in various environments. MAbs (2017) 9(4):664–79. doi: 10.1080/19420862.2017.1297909 PMC5419078 28387583

[22] Coppieters K Dreier T Silence K De Haard. H Lauwereys M Casteels P. Formatted antitumor necrosis factor α VHH proteins derived from camelids show superior potency and targeting to inflamed joints in a murine model of collagen-induced arthritis. Arthritis Rheumatism (2006) 54(6):1856–66. doi: 10.1002/art.21827 16736523

[23] Beirnaert E Desmyter A Spinelli S Lauwereys M Aarden L Dreier T. Bivalent llama single-domain antibody fragments against tumor necrosis factor have picomolar potencies due to intramolecular interactions. Front Immunol (2017) 8:867. doi: 10.3389/fimmu.2017.00867 28824615 PMC5534440

[24] Ishiwatari-Ogata C Kyuuma M Ogata H Yamakawa M Iwata K Ochi M. Ozoralizumab, a humanized anti-TNFα NANOBODY' compound, exhibits efficacy not only at the onset of arthritis in a human TNF transgenic mouse but also during secondary failure of administration of an anti-TNFα IgG. Front Immunol (2022) 13:853008. doi: 10.3389/fimmu.2022.853008 35273620 PMC8902368

[25] Takeuchi T Kawanishi M Nakanishi M Yamasaki H Tanaka Y. Phase II/III results of the anti-TNF multivalent NANOBODY® compound ‘ozoralizumab’ in patient with rheumatoid arthritis (OHZORA trial). Arthritis Rheumatol (2022) 74(11):1776–85. doi: 10.1002/art.42273 PMC9828347 35729713

[26] Goel N Stephens S. Certolizumab pegol. MAbs (2010) 2(2):137–47. doi: 10.4161/mabs.2.2.11271 PMC2840232 20190560

[27] Chemical Computing Group ULC. Molecular operating environment (MOE), 2020.0901. Montreal, QC, Canada: Chemical Computing Group ULC (2020).

[28] Abraham MJ Murtola T Schulz R Páll S Smith JC Hess B. GROMACS: High performance molecular simulations through multi-level parallelism from laptops to supercomputers. SoftwareX (2015) 1-2:19–25. doi: 10.1016/j.softx.2015.06.001

[29] Jorgensen WL Chandrasekhar J Madura JD Impey RW Klein ML. Comparison of simple potential functions for simulating liquid water. J Chem Physics. (1983) 79(2):926–35. doi: 10.1063/1.445869

[30] Hess B Kutzner C Spoel DVD Lindahl E. GROMACS 4:' algorithms for highly efficient, load-balanced, and scalable molecular simulation. J Chem Theory Computation (2008) 4(3):435–47. doi: 10.1021/ct700301q 26620784

[31] Berendsen HJC Postma JPM Gunsteren WFV DiNola A Haak JR. Molecular dynamics with coupling to an external bath. J Chem Physics (1984) 81(8):3684–90. doi: 10.1063/1.448118

[32] Bayly CI Cieplak P Cornell W Kollman PA. A well-behaved electrostatic potential based method using charge restraints for deriving atomic charges: the RESP model. J Phys Chem (1993) 97(40):10269–80. doi: 10.1021/j100142a004

[33] Nosé S. A molecular dynamics method for simulations in the canonical ensemble. Mol Physics (1984) 52(2):255–68. doi: 10.1080/00268978400101201

[34] Hoover WG. Canonical dynamics: Equilibrium phase-space distributions. Phys Rev A (1985) 31(3):1695–7. doi: 10.1103/PhysRevA.31.1695 9895674

[35] Parrinello M Rahman A. Polymorphic transitions in single crystals: a new molecular dynamics method. J Appl Physics (1981) 52(12):7182–90. doi: 10.1063/1.328693

[36] Behnen M Leschczyk C Möller S Batel T Klinger M Solbach W. Immobilized immune complexes induce neutrophil extracellular trap release by human neutrophil granulocytes *Via* FcγRIIIB and mac-1. J Immunol (2014) 193(4):1954–65. doi: 10.4049/jimmunol.1400478 25024378

[37] Coppieters K Dreier T Silence K De Haard H Lauwereys M Casteels P. Formatted anti-tumor necrosis factor alpha VHH proteins derived from camelids show superior potency and targeting to inflamed joints in a murine model of collagen-induced arthritis. Arthritis Rheum (2006) 54(6):1856–66. doi: 10.1002/art.21827 16736523

[38] Imada I Sato EF Miyamoto M Ichimori Y Minamiyama Y Konaka R. Analysis of reactive oxygen species generated by neutrophils using a chemiluminescence probe l-012. Analytical Biochem (1999) 271(1):53–8. doi: 10.1006/abio.1999.4107 10361004

[39] Jönsson F Mancardi DA Albanesi M Bruhns P. Neutrophils in local and systemic antibody-dependent inflammatory and anaphylactic reactions. J Leukocyte Biol (2013) 94(4):643–56. doi: 10.1189/jlb.1212623 23532517

[40] Klebanoff SJ. Myeloperoxidase: friend and foe. J Leukocyte Biol (2005) 77(5):598–625. doi: 10.1189/jlb.1204697 15689384

[41] Bradley PP Priebat DA Christensen RD Rothstein G. Measurement of cutaneous inflammation: estimation of neutrophil content with an enzyme marker. J Invest Dermatol (1982) 78(3):206–9. doi: 10.1111/1523-1747.ep12506462 6276474

[42] Carbo C Yuki K Demers M Wagner DD Shimaoka M. Isoflurane inhibits neutrophil recruitment in the cutaneous arthus reaction model. J Anesthesia (2013) 27(2):261–8. doi: 10.1007/s00540-012-1508-1 PMC3568683 23096126

[43] Schmidt RE Gessner JE. Fc receptors and their interaction with complement in autoimmunity. Immunol Letters (2005) 100(1):56–67. doi: 10.1016/j.imlet.2005.06.022 16125792

[44] Pallardy MJ Turbica I Biola-vidamment A. Why the immune system should be concerned by nanomaterials? Front Immunol (2017) 8:544. doi: 10.3389/fimmu.2017.00544 28555135 PMC5431153

[45] Mayadas TN Tsokos GC Tsuboi N. Mechanisms of immune ComplexMediated neutrophil recruitment and tissue injury. Circulation (2009) 120(20):2012–24. doi: 10.1161/CIRCULATIONAHA.108.771170 PMC2782878 19917895

[46] Jefferis R. Aggregation, immune complexes and immunogenicity. MAbs (2011) 3(6):503–4. doi: 10.4161/mabs.3.6.17611 PMC3242835 22123066

[47] Kim M Lee S Song M Yoo TH Lee B Kim Y. Comparative analyses of complex formation and binding sites between human tumor necrosis factor-alpha and its three antagonists elucidate their different neutralizing mechanisms. J Mol Biol (2007) 374(5):1374–88. doi: 10.1016/j.jmb.2007.10.034 17996896

[48] Hazenbos WL Gessner J Hofhuis FM Kuipers H Meyer D Heijnen IA. Impaired IgG-dependent anaphylaxis and arthus reaction in FcγRIII (CD16) deficient mice. Immunity (1996) 5(2):181–8. doi: 10.1016/S1074-7613(00)80494-X 8769481

[49] Williams TJ. Vascular permeability changes induced by complement-derived peptides. Agents Actions (1983) 13(5-6):451–5. doi: 10.1007/BF02176415 6195896

[50] Tian J Avalos AM Mao S Chen B Senthil K Wu H. Toll-like receptor 9dependent activation by DNA-containing immune complexes is mediated by HMGB1 and RAGE. Nat Immunol (2007) 8(5):487–96. doi: 10.1038/ni1457 17417641

[51] Joubert MK Hokom M Eakin C Zhou L Deshpande M Baker MP. Highly aggregated antibody therapeutics can enhance the in vitro innate and late-stage T-cell immune responses. J Biol Chem (2012) 287(30):25266–79. doi: 10.1074/jbc.M111.330902 PMC3408134 22584577

[52] Lundahl MLE Fogli S Colavita PE Scanlan EM. Aggregation of protein therapeutics enhances their immunogenicity: Causes and mitigation strategies. RSC Chem Biol (2021) 2(4):1004–20. doi: 10.1039/d1cb00067e PMC8341748 34458822

