# Supplementary figures and images for: Unique structure of ozoralizumab, a trivalent anti-TNFα NANOBODY® compound, offers the potential advantage of mitigating the risk of immune complex-induced inflammation
Source: Front Immunol. 2023 Apr 14;14:1149874. doi: 10.3389/fimmu.2023.1149874 (PMC10141648; doi:10.3389/fimmu.2023.1149874)

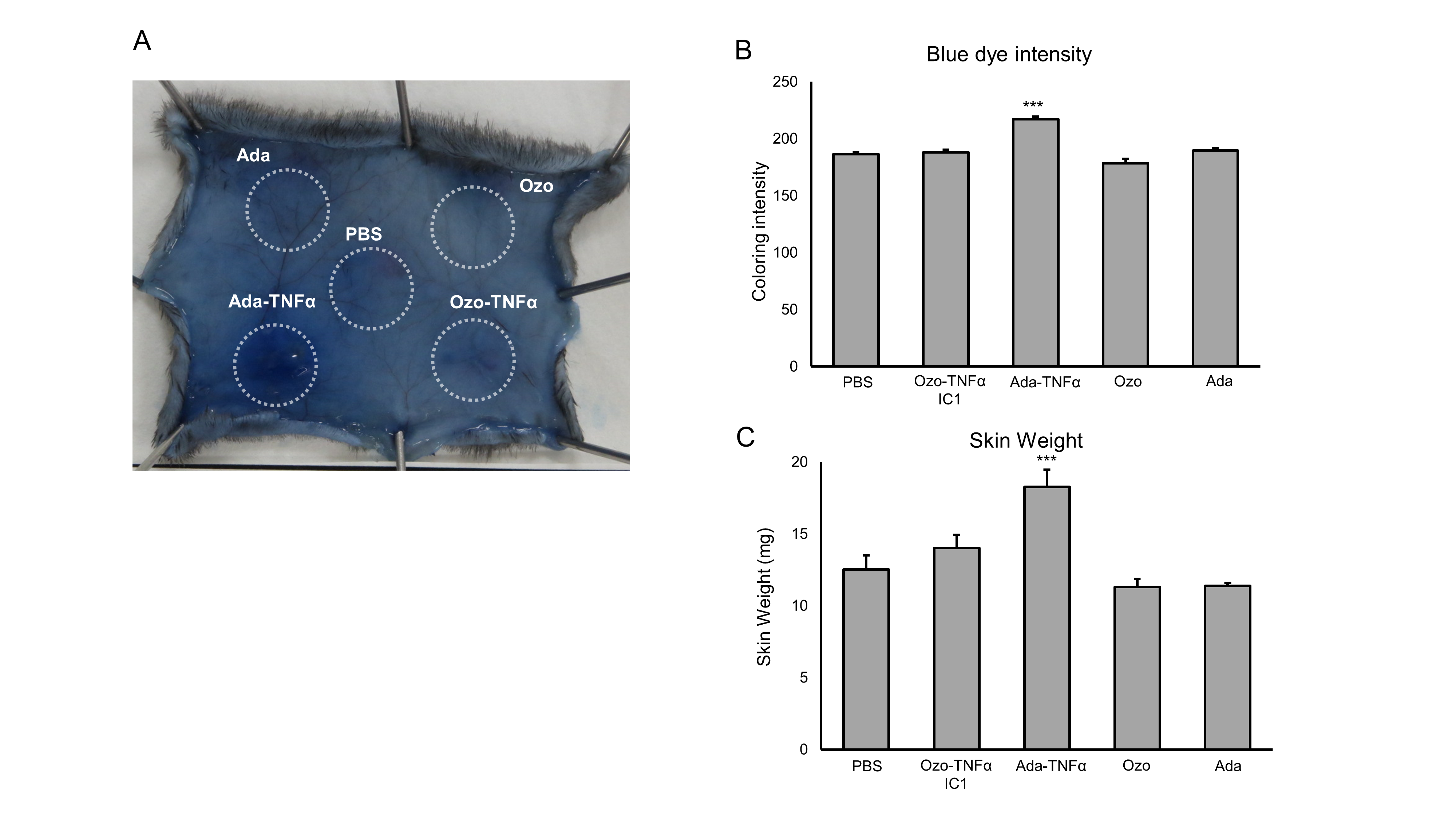

Supplement: Supplementary Figure 1 — Inflammatory responses in the skin of mice to intradermally injected with anti TNFα antibody alone and pre-incubated ICs. Mice were intradermally injected with PBS, ozoralizumab-TNFαICs (Ozo-TNFα IC1, the same conc/ratio as in Figure 4 ), adalimumab-TNFαICs (Ada-TNFα ICs, the same conc/ratio as in Figure 4 ), ozoralizumab (Ozo) and adalimumab (Ada) and then intravenously injected with 0.5% Evans blue. (A) Representative macroscopic appearance of the subcutaneous inflammation. The circles surround exuded Evans blue dye at each injection site. (B) Blue dye intensity was quantified with ImageJ software. (C) Skin weight of 6-mm skin punch biopsy specimens. n = 6 *** p <0.001 vs. PBS (Tukey’s test). [file Image_1.tif]

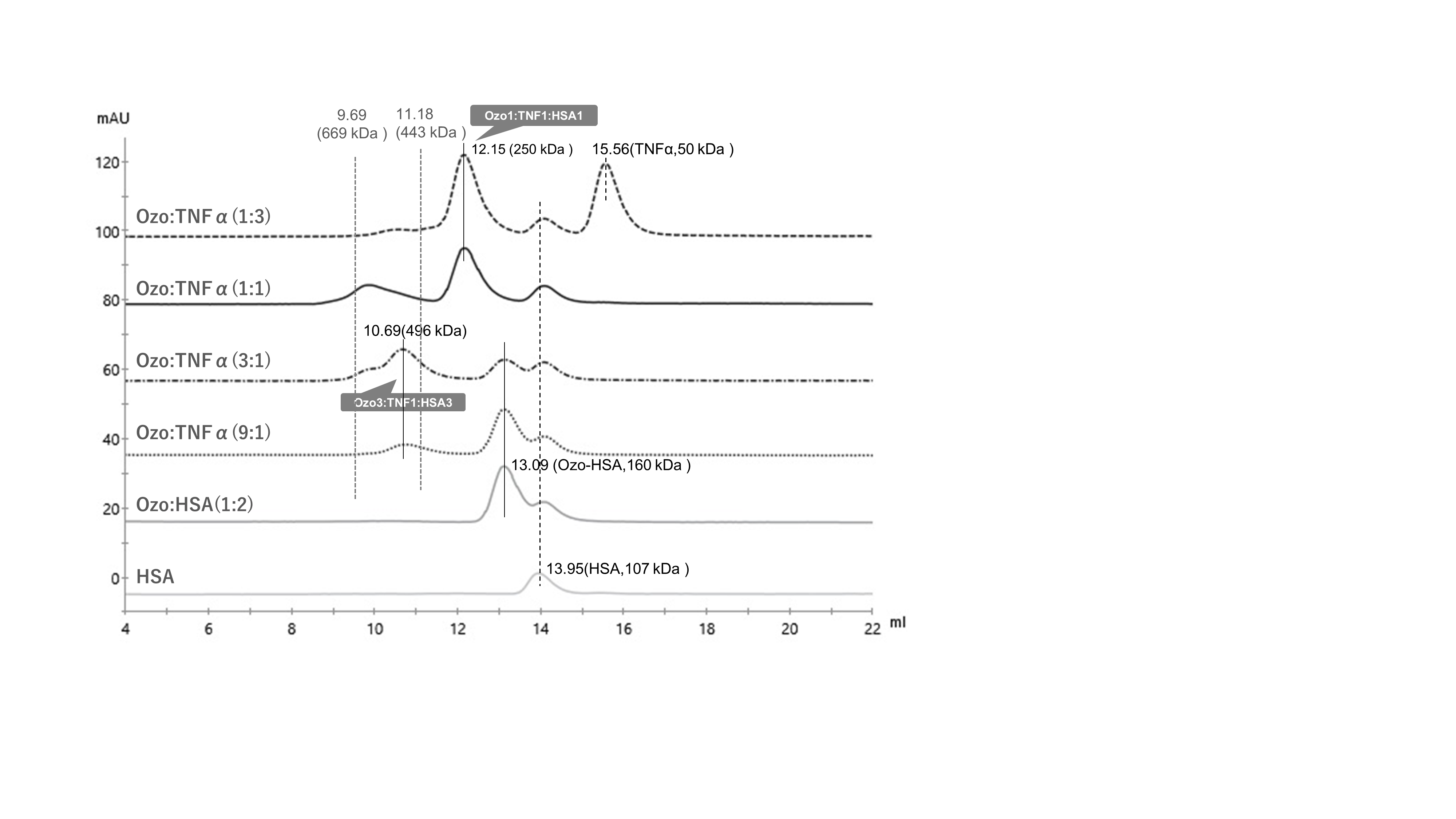

Supplement: Supplementary Figure 2 — Size Exclusion Chromatography (SEC) of immunocomplexes of ozoralizumab-TNFα-HSA. All molecular weights were estimated by calibration with molecular weight markers. [file Image_2.tif]

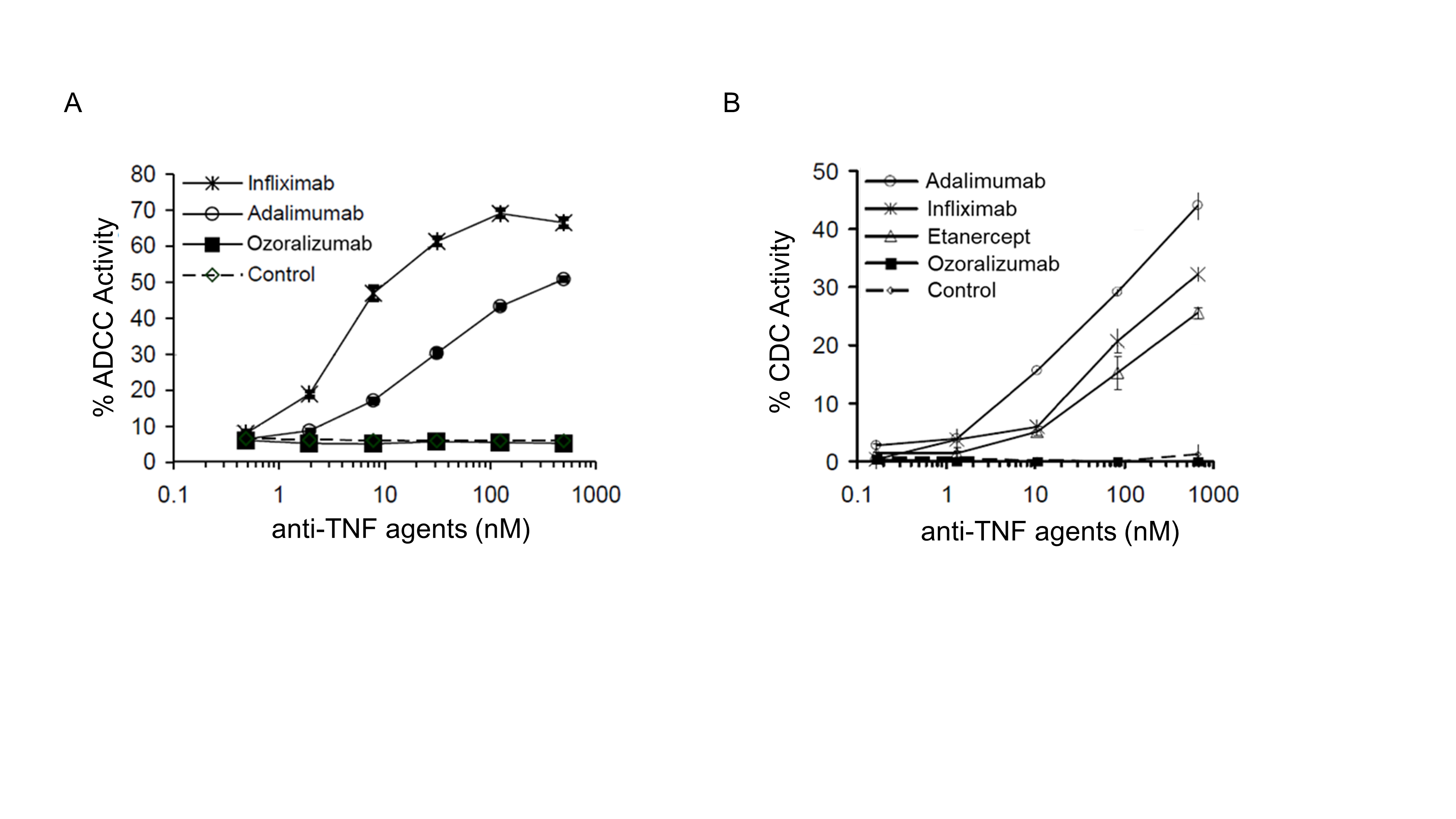

Supplement: Supplementary Figure 3 — (A) ADCC activity of infliximab, adalimumab, and a control IgG1 antibody (a human IgG1 antibody raised against the HIV envelop) were compared with ozoralizumab by using CFSE-labeled NS0-TNFD13 cells as targets and human NK cells as effectors. % ADCC activity values were calculated as the percentage of target cells that were 7AAD+. (B) CDC activity of etanercept, adalimumab, infliximab, and a control IgG1 antibody (a human IgG1 antibody raised against the HIV envelope) was compared with the CDC activity of ozoralizumab on the CHO-TNF NC2 line in vitro in the presence of baby rabbit complement. Cytotoxicity was assessed by measuring PI uptake of the dead cells. Values plotted are percentages of PI+ cells with the test and control subtracted from % PI- cells in the presence of complement alone. [file Image_3.tif]
